# Comparing the risk of cardiovascular diseases and all-cause mortality in four lifestyles with a combination of high/low physical activity and healthy/unhealthy diet: a prospective cohort study

**DOI:** 10.1186/s12966-022-01374-1

**Published:** 2022-11-16

**Authors:** Asma Kazemi, Najmeh Sasani, Zeinab Mokhtari, Abbas Keshtkar, Siavash Babajafari, Hossein Poustchi, Maryam Hashemian, Reza Malekzadeh

**Affiliations:** 1 Nutrition Research Center, Shiraz University of Medical Sciences, Shiraz, Iran; 2 Nutrition and Food Security Research Center, Isfahan University of Medical Sciences, Isfahan, Iran; 3 Department of Health Science Educational Development, School of Public Health, Tehran University of Medical Sciences, Tehran, Iran; 4 Nutrition Research Center, School of Nutrition and Food Sciences, Shiraz University of Medical Sciences, Shiraz, Iran; 5 Liver and Pancreatobiliary Diseases Research Center, Digestive Diseases Research Institute, Tehran University of Medical Sciences, Tehran, Iran; 6 Department of Biology, School of Arts and Sciences, Utica University, Utica, NY USA; 7 Digestive Oncology Research Center, Digestive Diseases Research Institute, Tehran University of Medical Sciences, Tehran, Iran

**Keywords:** Diet, Physical activity, All-cause mortality, Cardiovascular mortality

## Abstract

**Background:**

In this study, we assessed the risk of cardiovascular diseases (CVDs) and all-cause mortality in subjects having an only physical activity or a healthy diet or both compared to those having none of these healthy behaviors in the Golestan Cohort Study (GCS).

**Methods:**

A total of 50,045 participants aged ≥ 40 years were recruited from Golestan Province, Iran, from 2004 to 2008 and followed for a median of 13.9 years. Four lifestyles were compared: healthy diet and active (HDA), healthy diet but inactive (HDI), unhealthy diet but active (UDA), and unhealthy diet and inactive (UDI), with UDI being considered as the reference group. Diet quality was assessed by the Dietary Approaches to Stop Hypertension diet score, which was calculated based on a validated food frequency questionnaire. The primary outcomes were death from any cause and CVDs. Adjusted Cox models were used to estimate the hazards ratio (HR) and 95% confidence intervals (CI) for overall and CVDs mortality.

**Results:**

During 467,401 person-years of follow-up, 6,256 overall deaths and 2,043 confirmed CVDs deaths were reported. After adjustment for potential confounders, there was a significant lower risk for all-cause mortality in participants with both healthy behaviors (HR = 0.79, 95% CI = 0.73 to 0.86) or only one healthy behavior [HDI: HR = 0.84, 95% CI = 0.78 to 0.90)] and [UDI: HR = 0.91, 95% CI = 0.85 to 0.97] compared to those with both unhealthy behaviors. For CVDs mortality, the HDA lifestyle (HR = 0.74, 95%CI = 0.65 to 0.86), as well as the UDA lifestyle (HR = 0.83, 95%CI = 0.74 to 0.94) indicated a significant lower risk compared to the UDI lifestyle. The HDI lifestyle was not more effective than UDI.

**Conclusion:**

The greatest reduction in all-cause and CVDs mortality was related to the HDA. For all-cause mortality, both HDI and UDA lifestyles were associated with a decreased risk in comparison to UDI, but for CVDs mortality, only UDA but not HDI decreased the risk.

**Supplementary Information:**

The online version contains supplementary material available at 10.1186/s12966-022-01374-1.

## Key messages

• People who had a healthy diet and were physically active had the lowest risk of all-cause and cardiovascular mortality.
• The risk of cardiovascular mortality was reduced in people who had activity but didn’t adhere to a healthy diet while not in those who had a healthy diet but were not physically active.
• Adhering to one of the healthy behaviors, either physical activity or healthy diet, reduced the risk of all-cause mortality in non-obese subjects. However, for cardiovascular mortality risk, adhering to a healthy diet but not having physical activity was not different from an unhealthy diet and inactive lifestyle.

## Introduction

Cardiovascular diseases (CVDs) remain the leading cause of mortality in the world [1]. Poor quality diet and inactivity are well stablished behavioral risk factors of CVDs incidence and mortality [2, 3].  An unhealthy diet along with a lack of physical activity result in cardiometabolic risk factors including obesity, insulin resistance, hyperlipidemia and hypertension [4, 5]. On the other hand, healthy dietary patterns such as Mediterranean and vegetarian diets have been related to decreased CVD and its risk factors [6, 7]. Additionally, moderate to vigorous regular physical activity has considerable beneficial effects on CVDs prevention and treatment by reducing body weight, improving insulin sensitivity, hypertension, and atherogenic dyslipidemia [8].

Although the independent associations of physical activity and diet quality with CVDs and mortality are apparent, conjoint association of physical activity and diet with metabolic risk factors and mortality has been less studied. Results of few studies which have assessed the combined association of a healthy diet and physical activity on health outcomes and risk factors for CVDs [9–12], revealed that moderate and vigorous intensity physical activity together with adherence to a healthy diet is associated with better improvement in metabolic health disorders and health outcomes compared to either behavior alteration alone, but only one study indicated a significant interaction between physical activity and diet [12]. In addition, these studies verified if adhering to only one healthy behavior could be more beneficial than having no healthy behavior. Results were inconsistent; one study found that neither physical activity nor healthy diet were more beneficial than poor quality diet and sedentary lifestyle [12]. Two other studies demonstrated that even one healthy behavior could be of benefit for some of metabolic markers [9, 10].

A better understanding of the association between dietary intake and physical activity in relation to the mortality of cardiovascular diseases, especially in people who are not able to adhere to lifestyle changes in both nutrition and physical activity simultaneously, may help to identify appropriate and feasible strategies to reduce the burden of the diseases. Thus, the aim of this study was to evaluate the risk of CVDs and all-cause mortality in subjects having only physical activity or a healthy diet, or both, compared to those having none of these healthy behaviors in the Golestan Cohort Study (GCS).

## Materials and methods

### Study population and design

The design of the GCS has been explained in detail previously [13, 14]. Briefly, 50,045 participants (21,234 men and 28,811 women) aged 40–75 years were enrolled between 2004 and 2008 in rural and urban areas of the Golestan province in northeast Iran. All participants signed a written informed consent form at baseline. Participants having an energy intake of more than twice the interquartile range above the 75th percentile (3690 kcal/day for women and 4145 kcal/day for men) or less than twice the interquartile range below the 25th percentile of energy intake (300 kcal/day for women and 525 kcal/day for men) were excluded from the analyses since dietary data for the energy intake of more than these values could not be reliable. To minimize the possibility of recall bias, participants with self-reported history of diabetes, CVD, or cancer at time of enrollment were also excluded.

### Dietary assessment

Dietary intake was assessed using a 116-item semiquantitative food frequency questionnaire (FFQ) by face-to-face interview. The validity and reliability of the FFQ for this population have been explained previously [15]. Participants were asked about the frequency (daily, weekly, or monthly) of intake of each food item consumed during the previous year; portion sizes of food items reported in household measures were then converted into grams. To calculate the energy and nutrient intake of participants, the Iranian Food Composition Table [16] and the food composition tables of the United States Department of Agriculture (USDA) were applied [17].

We assessed the quality of the diet based on the Dietary Approaches to Stop Hypertension (DASH) score [18]. We calculated the DASH score based on Fung's method [19]. Fung's DASH score consists of eight components for a total of 40 points: seven food groups and one nutrient. A maximum of 5 points could be dedicated to each component. High intake of fruits, vegetables, low-fat dairy, whole grains, nuts, and legumes is emphasized such that the highest quintile received five points. Whereas for red and processed meat, sugar sweetened beverages, and sodium, limiting the intake is desirable, so the top quintile of these nutrients received one point. The overall scores were calculated by summing all the points.

### Physical activity

Method of physical activity assessment has been described in detail in a previous publication [20]. Intensity of physical activity was evaluated at four levels. Level 1 was defined as sedentary work that is often done while sitting (e.g., driving), level 2 was defined as standing or occasional walking (e.g., teaching). Level 3 activities consisted of indoor activities that resulted in a mild increase in heart rate and perspiration (e.g., housekeeping). Finally, the activities that caused a significant elevation in heart rate and sweating and are usually performed outdoors are considered level 4 activities. After that, the metabolic equivalents of each task per minute per week (METs.min/week) were calculated. Duration and frequency of individuals’ work activity were assessed by two questions: if the person worked every month throughout the year, and if intense physical activity was part of the daily work. Leisure time physical activity was also recorded by asking the number of minutes per day spent in light (e.g. walking), moderate (e.g. volleyball), and vigorous (e.g. running) activities.

### Outcome measures

Participants were followed up annually by telephone contact. Any report of cardiovascular disease deaths by participants’ family members followed by a GCS team visit to confirm the event by evaluating medical records or verbal autopsy. By reviewing verbal autopsy information and medical records, two internists independently determined the cause of death. In cases of disagreement between the two internists, a more experienced internist diagnosed the cause of death. The follow-up rate for a median duration of 13.9 years was over 99% of the study participants. The primary outcomes in this analysis were CVDs [myocardial infarction, coronary heart disease, stroke, or heart failure] and all-cause mortality.

### Assessment of potential confounders

At baseline, demographics, lifestyle behaviors, and other general information were collected using a structured questionnaire during face-to-face interviews. The questionnaire contained questions about sex, age, ethnicity, marital status, residential history, occupation, education, smoking habits, opium use, alcohol drinking, indicators of socioeconomic status, and medical history, including self-reported medically diagnosed diabetes mellitus, heart disease, stroke, and cancer. Potential confounders assessed in this analysis were age, sex, smoking status, opium and alcohol consumption, and wealth score. The wealth score was used as a substitute for socioeconomic status and was generated based on household appliances, vehicles, and other wealth-related characteristics using multiple correspondence analyses [21].

### Statistical analysis

Descriptive variables were reported as means ± standard deviation (SD) and percentages. Multivariable Cox proportional hazards regression models were used to estimate the hazard ratios (HRs) and 95% CIs for cardiovascular mortality in relation to four lifestyle groups, including A) healthy diet but physically inactive (HDI), B) unhealthy diet but physically active (UDA), C) unhealthy diet and physically inactive (UDI), and D) healthy diet and physically active (HDA), which was considered as the reference group. To compose these lifestyles, both physical activity and DASH-score were dichotomized at their medians. Participants who were in the first quantile of both physical activity and DASH-score were assigned to UDI; first quantile of physical activity and second quintile of DASH-score HDI; second quantile of physical activity and first DASH-score, UDA; and finally, the second quantile of both physical activity and DASH-score, HDA*.* We evaluated proportional hazards assumptions by testing the significance of time-dependent interaction terms for all variables. No evidence of violation was observed for all outcomes. Multivariable models were adjusted for age (years, continuous), gender (male, female), body mass index (BMI) (kg/m2, < 18.5, 18.5 to < 25, 25 to < 30, ≥ 30), education (formal, nonformal), smoking status (never, former, current), opium use (never, ever), wealth score ( tertiles), marital status (married, other), self-reported history of hypertension (yes, no), and total energy intake (kcal/d). To assess the potential interaction for age, gender, BMI, and wealth score, the likelihood ratio test was used, by multiplying lifestyle by each potential effect modifier. We also investigated the interaction of diet and physical activity as continuous variables in relation to CVD/all-cause mortality.

All statistical analyses were performed with STATA software (version 14, STATA Corp, College Station, TX, USA). Reported P values are two-sided. All *P* values < 0.05 were considered statistically significant.

## Results

**Fig. 1:**
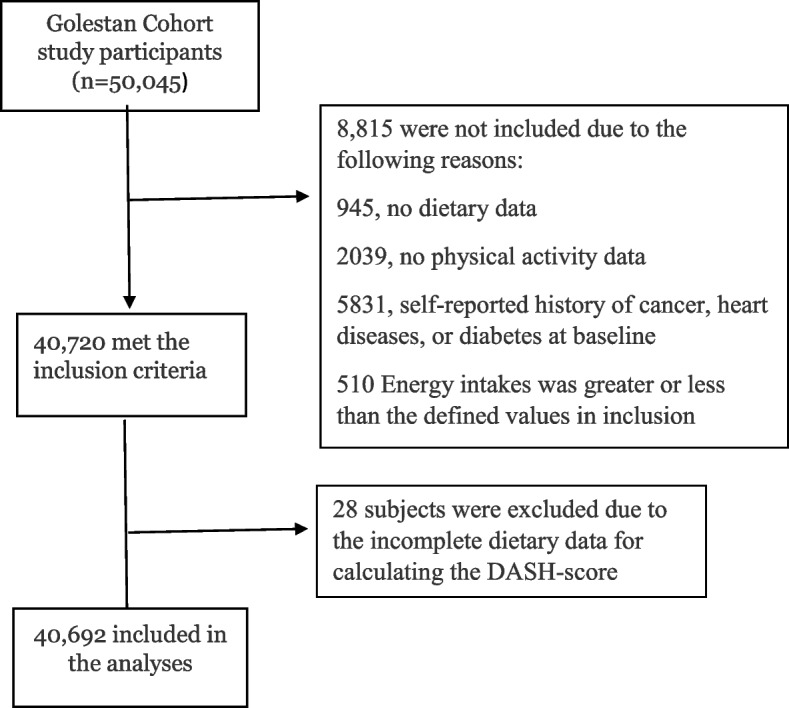
Flow chart of the participants of the present study

A total of 50,045 subjects were enrolled in the GCS. Of these, 40,692 (17,641 men and 23,051 women) individuals met the inclusion criteria and were included in the analyses (Fig. 1). During 467,401 person-years of follow up (2004–2021; total, 16.2 years; median, 13.9 years), 6,256 deaths and 2,043 confirmed deaths from CVDs (2836 both confirmed and non-confirmed) were documented in the Golestan Cohort Study. The loss to follow up rate was negligible (1%), which occurred mainly due to migration and losing access. The general characteristics of participants included in the analysis have been summarized in Table 1. The mean (SD) age of participants at baseline was 51.4(8.8) years, and 43% of them were male. Participants with inactive lifestyles (HDI and UDI) were more likely to be female and older. Participants with UDA were more likely to be opium users and smokers. Participants in HDI had the highest BMI and had a higher wealth score. A higher number of CVDs deaths was observed in males vs. females and in lower vs. higher wealth scores. The median DASH score was 22 out of 36 in the total population [25 and 20 in healthy (HDA and HDI) and unhealthy (UDA and UDI) lifestyles, respectively]. And the overall median physical activity was 10,414.5 [6660 in inactive lifestyles and 18,475 in active lifestyles]. Both all-cause and CVDs mortality represented a significant inverse relationship with physical activity and DASH-score (Table 2). We assessed the interaction between physical activity and DASH-score as continuous variables in relation to CVD and all-cause mortality and found no significant interaction (p values were 0.58 and 0.72 for CVD and all-cause mortality, respectively).

**Table 1:** General characteristics of Golestan Cohort Study participants according to lifestyles at baseline, (*n* = 40,692)

Variables	Unhealthy diet inactive	Unhealthy diet active	Healthy diet inactive	Healthy diet active
Participants, n (%)	11,426(28.08)	12,175 (29.92)	8,921(21.92)	8,170 (20.08)
Deaths (All cause), n	2,225	1,555	1,508	968
Age, years	53.43(9.42)	49.38(7.71)	53.16(9.44)	49.68(7.75)
Sex, male n (%)	3,130 (27.39)	7,100 (58.32)	2,613 (29.29)	4,798(58.73)
BMI, kg/m2	26.59 (5.75)	25.59(5.02)	27.57(5.57)	26.37 (5.0)
Energy, kcal/d	2018(542)	2231(567)	2144(538)	2299(560)
Wealth score, high (%)	44.6	41.25	62.77	57.18
Ever tobacco user ^c^ (%)	7.36	15.39	7.40	15.09
Ever opium user ^c^ (%)	16.16	18.72	13.07	16.98
Formal education, n (%)	80.69	68.01	66.17	56.0
Marital status, Married (%)	83.15	93.78	84.59	93.40
History of Hypertension (%)	20.05	10.20	21.39	11.87

Categories were reported as number or percentage of subjects. Continuous variables were reported by means and standard deviation

Abbreviation: *BMI* body mass index

All variables were statistically different across the lifestyle categories (P<0.001), ANCOVA for quantitative variables and χ 2 test for qualitative variables

**Table 2:** Hazard ratios and 95% confidence intervals for all-cause and CVDs mortality by physical activity and DASH score categories and high vs. low exposure in the Golestan Cohort Study

Outcome	Exposure	Q1	Q2	Q3	Q4	Q5	P trend	P interaction	High vs. low
All-cause mortality	Physical activity^1^	1.00 (reference)	0.84 (0.77, 0.91)	0.81 (0.75, 0.88)	0.78 (0.72, 0.84)	0.81 (0.75, 0.87)	< 0.001	< 0.001	0.85 (0.80, 0.90)
	DASH^2^	1.00 (reference)	0.95 (0.88, 1.02)	0.92 (0.86, 0.99)	0.90 (0.83, 0.97)	0.84 (0.77, 0.92)	< 0.001		0.92 (0.88, 0.97)
CVDs mortality	Physical activity^1^	1.00 (reference)	0.88 (0.77, 1.00)	0.79 (0.69, 0.91)	0.77 (0.67, 0.89)	0.80 (0.69, 0.91)	< 0.001	< 0.001	0.82 (0.74, 0.90)
	DASH^2^	1.00 (reference)	0.99 (0.88, 1.12)	0.94 (0.83, 1.06)	0.92 (0.80, 1.05)	0.85 (0.73, 0.99)	0.006		0.91 (0.83, 0.99)

Abbreviations: *CVDs* cardiovascular diseases, *DASH* Dietary Approaches to Stop Hypertension diet, *Q* quintile

^1^Adjusted for age, gender, BMI, energy intake, cigarette, opioid use, education, marital status, history of hypertension, DASH score, and wealth score

^2^Adjusted for age, gender, BMI, energy intake, cigarette, opioid use, education, marital status, history of hypertension, and wealth score, wealth score, and physical activity

In comparing the four lifestyles, there was a significant lower risk for all-cause mortality in participants with both healthy behaviors (HR = 0.79, 95% CI = 0.73 to 0.86) or one healthy behavior [HDI: HR = 0.84, 95% CI = 0.78 to 0.90)] and [UDA: HR = 0.91, 95% CI = 0.85 to 0.97] compared to those with both unhealthy behaviors (UDI) (Table 3). We also examined whether the association between lifestyles and all-cause mortality risk differed according to BMI, gender, age, and wealth score. There was a significant interaction between risk associations and BMI subgroups (*p* = 0.008). For other potential effect modifiers, the interaction was not significant. For all three lifestyles (having both or only one of the healthy behaviors) versus the UDI lifestyle, the reduction in mortality risk was significant among participants who had BMI < 30, but not among participants with BMI ≥ 30.

**Table 3:** Hazard ratios for all-cause mortality in relation to four lifestyles in the Golestan Cohort Study

Model	Unhealthy diet and inactive	Unhealthy diet but Active	Healthy Diet but Inactive	Healthy diet and active
**Total (case/n: 6,256/ 40,692)**				
Case/ n	2,225/11,426	1,555/12,175	1,508/8,921	968/8,170
Age and sex adjusted HR	1.00	0.82 (0.77, 0.88)	0.84 (0.79, 0.90)	0.73 (0.68, 0.79)
Fully adjusted HR^1^	1.00	0.84 (0.78, 0.90)	0.91 (0.85, 0.97)	0.79 (0.73, 0.86)
**Male (3,616/ 17,661)**				
Case/ n	986/3130	1215/7,100	673/2,613	738/4,798
Age adjusted HR	1.00	0.85 (0.78, 0.93)	0.85 (0.77, 0.94)	0.74 (0.67, 0.81)
Fully adjusted HR^2^	1.00	0.86 (0.79, 0.94)	0.93 (0.84, 1.03)	0.80 (0.72, 0.88)
**Female (2,647/ 23,063)**				
Case/ n	1239/8,296	340/5,075	835/6,308	230/3,372
Age adjusted HR	1.00	0.76 (0.67, 0.86)	0.83 (0.76, 0.91)	0.73 (0.63, 0.84)
Fully adjusted HR^2^	1.00	0.76 (0.67, 0.86)	0.90 (0.83, 0.99)	0.81 (0.71, 0.94)
**BMI < 30 (5,091/ 30,837)**				
Case/ n	1,827/8,481	1,321/9,908	1,158/6,120	781/6,328
Age and sex adjusted HR	1.00	0.80 (0.74, 0.87)	0.84 (0.78, 0.90)	0.71 (0.65, 0.77)
Fully adjusted HR^3^	1.00	0.82 (0.76, 0.89)	0.90 (0.83, 0.97)	0.77 (0.71, 0.85)
**BMI ≥ 30 (1,169/9,850)**				
Case/ n	396/2,943	234/2,267	349/2,800	187/1,840
Age and sex adjusted HR	1.00	0.93 (0.78, 1.10)	0.88 (0.76, 1.02)	0.77 (0.69, 0.99)
Fully adjusted HR^3^	1.00	0.95 (0.80, 1.13)	0.98 (0.85, 1.14)	0.90 (0.74, 1.07)

Abbreviations: *BMI* body mass index, *HR* hazard ratio

^1^Adjusted for age, gender, BMI, energy intake, cigarette, opioid use, education, marital status, history of hypertension, and wealth score

^2^Adjusted for age, BMI, energy intake, cigarette, opioid use, education, marital status, history of hypertension, and wealth score

^3^Adjusted for age, gender, energy intake, cigarette, opioid use, education, marital status, history of hypertension, and wealth score

For CVDs mortality, participants with the HDA lifestyle (HR = 0.74, 95%CI = 0.65 to 0.86), as well as participants with the UDA lifestyle (HR = 0.83, 95%CI = 0.74 to 0.94) had a significantly lower risk compared to those with the UDI lifestyle in overall population; the HDI lifestyle was not associated with the reduced risk compared to the UDI. There was a significant interaction between risk associations and BMI subgroups (*p* < 0.001) and gender (*p* = 0.004). No interaction was observed for other effect modifiers. For the HDA and UDA lifestyles versus UDI, the reduction in CVD mortality risk was significant and greater among females and participants with BMI < 30. In males, only the HDA was associated with reduced risk compared to the UDI. In subjects with BMI ≥ 30, none of the three groups reduced risk of CVDs mortality compared to the UDI. The HDI lifestyle was associated with the reduced risk compared to the UDI in none of the subgroups. Results of CVDs mortality have been presented in Table 4.

**Table 4:** Hazard ratios for cardiovascular mortality in relation to four lifestyles in Golestan Cohort Study

Model	Unhealthy diet and inactive	Unhealthy diet but Active	Healthy Diet but Inactive	Healthy diet and active
**Total (2,043/ 40,692)**				
Case/n	757/ 11,427	480/12177	522/8922	284/8170
Age and sex adjusted HR	1.00	0.81 (0.72, 0.91)	0.88 (0.78, 0.98)	0.68 (0.59, 0.78)
Fully adjusted HR^1^	1.00	0.83 (0.74, 0.94)	0.94 (0.84, 1.05)	0.74 (0.65, 0.86)
**Male (1,152/ 17,626)**				
Case/ n	319/ 3131	387/7101	230/2613	216/4798
Age adjusted HR	1.00	0.88 (0.76, 1.03)	0.93 (0.78, 1.1)	0.70 (0.58, 0.83)
Fully adjusted HR^2^	1.00	0.90 (0.77, 1.06)	0.99 0.83, 1.17	0.74 (0.62, 0.89)
**Female (891 / 23,019)**				
Case/ n	438/ 8,296	93/ 5,076	292/ 6,309	68/ 3,372
Age adjusted HR	1.00	0.64 (0.51, 0.80)	0.84 (0.72, 0.97)	0.67 (0.51, 0.86)
Fully adjusted HR^2^	1.00	0.65 (0.52, 0.82)	0.90 (0.77, 1.05)	0.75 (0.58, 0.97)
**BMI < 30(1,620/ 30,837)**				
Case/ n	600/8,481	386/9,908	414/6,120	220/6,328
Age and sex adjusted HR	1.00	0.77 (0.67, 0.88)	0.93 (0.82, 1.05)	0.66 (0.56, 0.77)
Fully adjusted HR^3^	1.00	0.79 (0.69, 0.91)	0.98 (0.86, 1.11)	0.72 (0.61, 0.84)
**BMI ≥ 30 (421/ 9,850)**				
Case/ n	155/2,943	94/2,267	108/2,800	64/,840
Age and sex adjusted HR	1.00	1.02 (0.77, 1.34)	0.72 (0.56, 0.92)	0.77 (0.56, 1.04)
Fully adjusted HR^3^	1.00	1.05 (0.79, 1.38)	0.81 (0.63, 1.04)	0.85 (0.63, 1.16)

Abbreviations: *BMI*, body mass index, *HR* hazard ratio

^1^Adjusted for age, gender, BMI, energy intake, cigarette, opioid use, education, marital status, history of hypertension, and wealth score

^2^Adjusted for age, BMI, energy intake, cigarette, opioid use, education, marital status, history of hypertension, and wealth score

^3^Adjusted for age, gender, energy intake, cigarette, opioid use, education, marital status, history of hypertension, and wealth score

## Discussion

Our study demonstrated a reduction of 21, 16, and 9 percentage in all-cause mortality in participants who engaged in both healthy behaviors, only physical activity, and only a healthy diet, when compared to those with both unhealthy behaviors, respectively. For CVDs mortality, participants with HDA and UDA lifestyles showed a 26 and 17% decrease, respectively compared to the UDI lifestyle, whereas HDI was not associated with risk of CVD mortality in this study. Additionally, an interaction with BMI was observed for both all-cause and CVD mortality, such that the reduction in all-cause and CVD mortality remained significant in non-obese subjects, but it was not significant in obese participants for none of the lifestyles. For CVD mortality, UDA lifestyle was associated with risk reduction only in females.

The findings could be interpreted in several ways. First, the combination of healthy diet and physical activity was associated with a greater reduction in all-cause or CVD mortality than either behavior change alone, suggesting the necessity to include physical activity in combination with diet to elicit the highest likelihood of risk reduction. Second, adhering to only one of the healthy behaviors could be successful in decreasing the risk of all-cause mortality. Whereas, adhering to a healthy diet without engaging in physical activity was not associated with the risk of CVD mortality. Additionally, the benefits of healthy behaviors (either one or both) in nonobese individuals could be explained by the fact that obesity, one of the leading causes of death, may attenuate the advantage of healthy behaviors in increasing life expectancy or delaying death in people with higher BMI.

Several studies have assessed conjoint assessment of physical activity and diet in relation to various health outcomes. Ricci et al. [22] compared four lifestyles consisting of various combinations of sedentariness and diet quality in a sample of 2,473 US adult participants followed for a median period of 5.6 years. Diet quality was assessed by the amount of fiber and micronutrients intake. They observed an inverse all-cause mortality risk in the range of 35% to 41% and a reduced CVDs mortality risk in the range of 32% to 61% when low sedentariness and high dietary fiber and vitamin intakes were mutually present. In low sedentariness and low dietary fiber and vitamin intakes, a significant reduction was detected in the risk of all-cause mortality but not CVDs mortality. In those with high sedentariness and high dietary fiber and vitamin intakes, no significant change was found in both all-cause and CVDs mortality. The results of this study are similar to ours in that the UDA lifestyle was associated, while, the HDI was not associated with CVD mortality.

In a cross-sectional study of 2,629 children and adolescents in the US, the relationship between the four lifestyles and CVDs risk factors was investigated [23]. Children with an HDA lifestyle had significantly lower waist circumference, C-reactive protein (CRP), and triglycerides than children with UDI lifestyle. Children with an HDA lifestyle had significantly lower CRP and total cholesterol levels than those with UDA; there were no significant differences in biomarker levels among children with an HDA lifestyle and those with an HDI lifestyle. For adolescents, no significant associations were observed.

In another cross-sectional study, in 1,513 European adolescents, association of these four lifestyles with CVDs risk score was evaluated [24]. CVDs risk score was composed by mean of z-score from cardiorespiratory fitness, the sum of four skinfolds, triglycerides, total cholesterol/high-density lipoprotein cholesterol ratio, and insulin resistance. The HDA group had the lowest CVDs risk score and the UDI group had the highest. HDA had a lower CVDs risk score than inactive adolescents with either a healthy or unhealthy diet, but the difference between HDA and UDA was not significant.

Our study has some limitations. The first one pertains to the potential weaknesses of FFQs, such as measurement error, however, in the GCS, four repetitions of the FFQ and twelve 24-h recalls were used to validate the FFQ. Another possible limitation is represented using self-reported data on physical activity. The third limitation was that the data on dietary intake was collected only at baseline, which may not reflect long-term variations in intakes and changes in dietary habits over the course of follow-up. Furthermore, it should be noted that due to the observational nature of the study, the observed associations don’t reflect cause and effect. Finally, our results may not be generalizable to other populations since most of the participants had Turkmen ethnicity. Our study also has several strengths, including the large sample size, the prospective design, long-term follow-up, and a low rate of loss to follow-up.

## Conclusion

Our findings indicate that in this population, the greatest reduction in all-cause and CVDs mortality was observed in subjects adhering to both healthy behaviors, suggesting the necessity to include physical activity in combination with diet to elicit the highest likelihood of risk reduction. For all-cause mortality, adhering to only healthy behavior was also associated with a decreased risk in comparison to unhealthy inactive lifestyle (the effect of UDA was greater than HDI), but for CVD mortality, only UDA but not HDI decreased the risk, which suggests the greater importance of physical activity vs. diet in reducing CVDs mortality risk.

## Supplementary Information

**Additional file 1.**

## Data Availability

The datasets used and/or analyzed during the current study are available from the 6th author (HP) on reasonable request.

## Abbreviations

BMI: Body mass index
CI: Confidence interval
CVD: Cardiovascular disease
DASH: Dietary Approaches to Stop Hypertension
FFQ: Food frequency questionnaire
GCS: Golestan Cohort Study
HDA: Healthy diet active
HDI: Healthy diet inactive
HR: Hazard ratio
Kcal: Kilocalorie
MET: Metabolic equivalents
SD: Standard deviation
UDA: Unhealthy diet inactive
UDI: Unhealthy diet inactive
USDA: United States Department of Agriculture
